# Point-of-Care Ultrasound Assessment of Pulmonary and Venous Congestion in Patients with Liver Cirrhosis and Acute Kidney Injury Receiving Albumin: An Exploratory Prospective Study from a Resource-Limited Tertiary Care Center in Western Mexico

**DOI:** 10.3390/medicina62081461

**Published:** 2026-07-28

**Authors:** Brian Rafael Rubio-Mora, Mario Alberto Ochoa-Rodríguez, Mauricio Alfredo Ambriz-Alarcón, Ernesto Alejandro Lozano-Sabido, Héctor Meugniot-García, Diego Moisés Jiménez-Pérez, Álvaro Ismael Calleros-Camarena, Sol Ramírez-Ochoa, Berenice Vicente-Hernández, Gabino Cervantes-Guevara, Enrique Rabago-Solorio, Enrique Cervantes-Perez

**Affiliations:** 1 Departamento de Medicina Interna, Hospital de Especialidades, Centro Médico Nacional de Occidente “Lic. Ignacio García Téllez”, Instituto Mexicano del Seguro Social, Guadalajara 44350, Jalisco, Mexico; brianrrm@gmail.com (B.R.R.-M.); marioa8ar@hotmail.com (M.A.O.-R.); mambriz@hcg.gob.mx (M.A.A.-A.); eals06@gmail.com (E.A.L.-S.); 2 Departamento de Urgencias Adultos, Hospital Civil de Guadalajara “Fray Antonio Alcalde”, Guadalajara 44280, Jalisco, Mexico; 3 División de Medicina, Hospital Civil de Guadalajara “Fray Antonio Alcalde”, Guadalajara 44280, Jalisco, Mexico; 4 Departamento de Medicina Interna, Hospital San Javier, Guadalajara 44670, Jalisco, Mexico; hecmg25@gmail.com (H.M.-G.); dm.jimenezp@outlook.com (D.M.J.-P.); 5 Centro de Enfermedades Digestivas y Hepáticas, Hospital Puerta de Hierro Andares, Zapopan 45116, Jalisco, Mexico; alvaro.i.calleros.c@gmail.com; 6 Departamento de Medicina Interna, Hospital Civil de Guadalajara “Fray Antonio Alcalde”, Centro Universitario de Ciencias de la Salud, Universidad de Guadalajara, Guadalajara 44280, Jalisco, Mexico; sramirez@hcg.gob.mx (S.R.-O.); dra.berenicevicente@gmail.com (B.V.-H.); 7 Departamento de Gastroenterología, Hospital Civil de Guadalajara Fray Antonio Alcalde, Guadalajara 44280, Jalisco, Mexico; gabino_guevara@hotmail.com; 8 Departamento de Bienestar y Desarrollo Sustentable, Centro Universitario del Norte, Universidad de Guadalajara, Colotlán 46200, Jalisco, Mexico; erabagoss@hotmail.com; 9 Departamento de Clínicas, Centro Universitario de Tlajomulco, Universidad de Guadalajara, Tlajomulco de Zuñiga 45641, Jalisco, Mexico

**Keywords:** cirrhosis, acute kidney injury, point-of-care ultrasound, albumin, pulmonary congestion, venous congestion, resource-limited settings

## Abstract

*Background and Objectives*: Patients with cirrhosis and acute kidney injury (AKI) frequently receive albumin, although plasma volume expansion may contribute to pulmonary or venous congestion. Point-of-care ultrasound (POCUS) may complement bedside assessment when formal echocardiography or advanced hemodynamic monitoring is not immediately available. This study evaluated baseline pulmonary and venous congestion using POCUS in patients with cirrhosis and AKI receiving albumin therapy. *Materials and Methods*: This exploratory prospective cohort included adults with cirrhosis, ascites, and ICA-AKI stage 1B or higher who were managed under an institutional protocol prescribing intravenous 20% or 25% albumin at 1 g/kg/day for two consecutive days, capped at 100 g/day. Before albumin administration, B-line-defined pulmonary congestion was assessed using a 28-site lung protocol, and IVC-defined venous congestion was assessed using inferior vena cava (IVC) diameter and collapsibility. Serum creatinine was recorded within 12 h before treatment and 48 h after initiation. Complete renal response was defined as serum creatinine within 0.3 mg/dL of the pre-AKI baseline; partial response was defined as regression by at least one ICA-AKI stage without complete response. *Results*: Twenty-two patients were included. B-line-defined pulmonary congestion was present in 18/22 (81.8%). IVC assessment was technically evaluable in 20 patients, of whom 6/20 (30.0%) met the criteria for IVC-defined venous congestion. The Hodges–Lehmann estimate of the median paired creatinine difference was +0.03 mg/dL (95% CI, −0.52 to 1.31) without pulmonary congestion and −0.33 mg/dL (95% CI, −0.67 to −0.20) with pulmonary congestion. Renal response occurred in 1/4 (25.0%) and 12/18 (66.7%), respectively (two-sided Fisher exact *p* = 0.264). Among patients with evaluable IVC examinations, renal response occurred in 8/14 (57.1%) without and 3/6 (50.0%) with IVC-defined venous congestion (*p* = 1.000). *Conclusions*: Baseline POCUS frequently identified ultrasound-defined congestion, but congestion status did not clearly distinguish short-term renal response. These exploratory findings support the feasibility of bedside POCUS phenotyping but do not establish that congestion modifies albumin response or clinical outcomes.

## 1. Introduction

Patients with cirrhosis are at high risk of developing acute kidney injury (AKI), particularly during hospitalization and critical illness [1]. AKI in this population is multifactorial and may involve portal hypertension, splanchnic vasodilation, reduced effective arterial blood volume, infection, gastrointestinal bleeding, nephrotoxic exposure, diuretic-related fluid losses, acute tubular injury, and hepatorenal syndrome-AKI (HRS-AKI) [2,3,4].

Albumin infusion is a key component of the initial evaluation and management of AKI in patients with cirrhosis, particularly when HRS-AKI is suspected. Treatment commonly includes intravenous 20% or 25% albumin at 1 g/kg/day for two consecutive days, capped at 100 g/day, together with withdrawal of diuretics, beta-blockers, and potentially nephrotoxic medications when clinically appropriate [2,4]. However, plasma volume expansion may increase cardiac filling pressures and contribute to pulmonary or venous congestion in susceptible patients [5,6]. Fluid overload and congestion have been associated with adverse outcomes in critically ill populations [7,8].

Point-of-care ultrasound (POCUS) provides rapid, non-invasive bedside information that may complement physical examination by characterizing pulmonary B-line burden and inferior vena cava (IVC) parameters [5,9,10,11]. This approach may be particularly useful when immediate access to formal echocardiography, advanced hemodynamic monitoring, or specialized imaging is limited or delayed. Nevertheless, B-lines, extravascular lung water, pulmonary edema, and generalized volume overload are related but non-equivalent concepts and should not be used interchangeably.

Evidence regarding baseline POCUS assessment of pulmonary and venous congestion in patients with cirrhosis and AKI receiving albumin remains limited [12,13,14]. Therefore, this exploratory prospective study aimed to evaluate B-line-defined pulmonary congestion and IVC-defined venous congestion before albumin administration in patients with cirrhosis and AKI at a tertiary referral center in western Mexico.

## 2. Materials and Methods

Study Design and Setting: This was an exploratory prospective cohort study conducted in the Gastroenterology Service of the Hospital de Especialidades, Centro Médico Nacional de Occidente “Lic. Ignacio García Téllez”, Instituto Mexicano del Seguro Social, a tertiary referral center in western Mexico. In the study setting, immediate bedside access to formal echocardiography, advanced hemodynamic monitoring, and specialized imaging could be limited or delayed. The study included patients with cirrhosis and acute kidney injury who were prescribed albumin infusion therapy between 1 July 2022 and 30 November 2022. Given the exploratory nature of the study, a formal sample size calculation was not performed.

Study Population: Patients were eligible if they met the following inclusion criteria: individuals of either sex, aged 18 to 80 years, with ascites, decompensated cirrhosis, spontaneous breathing, and ICA-AKI stage 1B or higher. AKI staging was based on the International Club of Ascites classification. ICA-AKI stage 1B was defined as an increase in serum creatinine >0.3 mg/dL (26.5 µmol/L) or an increase of 1.5–2.0 times from pre-AKI baseline, with an absolute serum creatinine level ≥1.5 mg/dL. Patients with higher AKI stages were also eligible.

Patients were excluded if pre-AKI baseline creatinine was unknown, if they had severe valvular heart disease, including severe valvular insufficiency or stenosis, active variceal hemorrhage, requirement for mechanical ventilatory support, pre-existing chronic kidney disease corresponding to KDIGO stage 4 or 5, tense ascites at the time of POCUS evaluation, or if they declined participation.

Data Collection: Clinical and laboratory data were collected from electronic medical records. The variables recorded included age, sex, pre-AKI baseline serum creatinine, pre-albumin serum creatinine, and serum creatinine 48 h after albumin initiation. Pre-AKI baseline creatinine was defined as the lowest serum creatinine value documented within the previous 3 months and was used for AKI staging and renal response classification. Pre-albumin creatinine was defined as the serum creatinine value obtained within 12 h before the start of albumin infusion and was used to describe the paired change over 48 h. These laboratory values were obtained as part of routine clinical care, and the research team recorded the results in a structured data collection form before entry into a study-specific Microsoft Excel spreadsheet.

Albumin Infusion Protocol: Albumin infusion was prescribed according to the institutional protocol for patients with cirrhosis and AKI. The protocol specified intravenous 20% or 25% human albumin at 1 g/kg/day for two consecutive days, capped at 100 g/day. Withdrawal of potentially nephrotoxic medications and other treatment adjustments were determined by the treating clinical team. Albumin administration was part of routine clinical care and was not assigned by the research team. Individual administered doses, cumulative dose, and total infusion volume were not systematically collected and could not be analyzed.

Point-of-Care Ultrasound Assessment: After confirmation of eligibility and before albumin administration, all patients underwent point-of-care ultrasound (POCUS) while breathing spontaneously in the supine position. The protocol was designed to assess B-line-defined pulmonary congestion and IVC-defined venous congestion at the bedside.

For lung ultrasound, the right and left hemithoraces were examined from the second to the fourth intercostal spaces (and the fifth intercostal space on the right) along the parasternal, midclavicular, anterior axillary, and midaxillary lines, providing 28 standardized scanning sites [15,16]. B-lines were defined as vertical reverberation artifacts arising from the pleural line, extending to the bottom of the image without fading, and moving synchronously with pleural sliding [16]. The number of B-lines at each site was recorded and summed. A total of at least six B-lines was operationally defined as B-line-defined pulmonary congestion. Severity was categorized as mild (6–15 B-lines), moderate (16–30 B-lines), or severe (>30 B-lines), according to previously proposed classifications [15,17]. This definition reflects sonographic B-line burden and should not be interpreted as equivalent to clinically confirmed pulmonary edema or generalized volume overload.

For IVC assessment, a subxiphoid view was obtained through the liver, and the IVC was visualized in the long axis. Maximum and minimum IVC diameters during spontaneous respiration were measured approximately 2 cm caudal to the right atrium-IVC junction. The collapsibility index was calculated as [(maximum diameter − minimum diameter)/maximum diameter] × 100. IVC-defined venous congestion was operationally defined as a maximum IVC diameter of at least 2.0 cm combined with a collapsibility index <50% [18]. When the IVC could not be reliably visualized because of anatomical limitations or an inadequate acoustic window, the participant was considered non-evaluable for venous congestion and was excluded only from venous congestion analyses.

All examinations were performed with a SonoScape E1 portable ultrasound system equipped with linear and convex transducers (SonoScape Medical Corp., Shenzhen, China), and images were interpreted at the bedside. All examinations were performed by a single trained physician operator with five years of POCUS experience. For intraobserver quality control, 20 stored lung ultrasound loops were re-evaluated by the same operator, yielding an intraclass correlation coefficient of 0.90 for B-line counts. No interobserver reproducibility assessment was performed.

Renal Response Definition: Renal response was evaluated using the change from the pre-AKI baseline serum creatinine to the value measured 48 h after albumin initiation, in accordance with the International Club of Ascites criteria [4]. Complete response was defined as return of serum creatinine to a value within 0.3 mg/dL of the pre-AKI baseline. Partial response was defined as regression by at least one ICA-AKI stage, with the final serum creatinine remaining at least 0.3 mg/dL above the pre-AKI baseline. Patients meeting either complete- or partial-response criteria were operationally classified as renal responders for group comparisons.

Statistical Analysis: Descriptive statistics were used to characterize the study population. Quantitative variables are presented as mean ± standard deviation (SD), and categorical variables as frequencies and percentages. Denominators were defined according to the evaluable population for each POCUS domain. Pulmonary congestion analyses included all patients with lung ultrasound assessment, whereas venous congestion analyses included only patients in whom IVC diameter and collapsibility could be reliably assessed.

Comparisons of renal responder proportions between congestion groups were performed using the two-sided Fisher exact test. The distribution of paired serum creatinine differences was assessed using the Shapiro–Wilk test. Because the normality assumption was not met, changes between the pre-albumin serum creatinine value and the value measured 48 h after albumin initiation were evaluated using the exact Wilcoxon signed-rank test. The magnitude and precision of paired changes were summarized using the Hodges–Lehmann estimate of the median paired difference with its corresponding exact 95% confidence interval. Analyses according to pulmonary congestion severity were limited to descriptive summaries because of the very small subgroup sizes; no formal hypothesis testing or multiplicity adjustment was performed for these categories. All analyses were exploratory, and *p*-values were interpreted descriptively rather than as confirmatory evidence. Statistical analyses were performed using IBM SPSS Statistics version 26 (IBM Corp., Armonk, NY, USA). Exact Wilcoxon analyses and Hodges–Lehmann estimates were obtained using GraphPad Prism version 8 (GraphPad Software, San Diego, CA, USA).

Ethical Considerations: The study was conducted in accordance with the Declaration of Helsinki and Good Clinical Practice guidelines. The study protocol was approved by the Clinical Research and Bioethics Committee of the Hospital de Especialidades, Centro Médico Nacional “Lic. Ignacio García Téllez” (approval code: R-2022-1301-239; date of approval: 15 June 2022). All participants provided written informed consent before undergoing any study-related procedures.

## 3. Results

**Figure 1:**
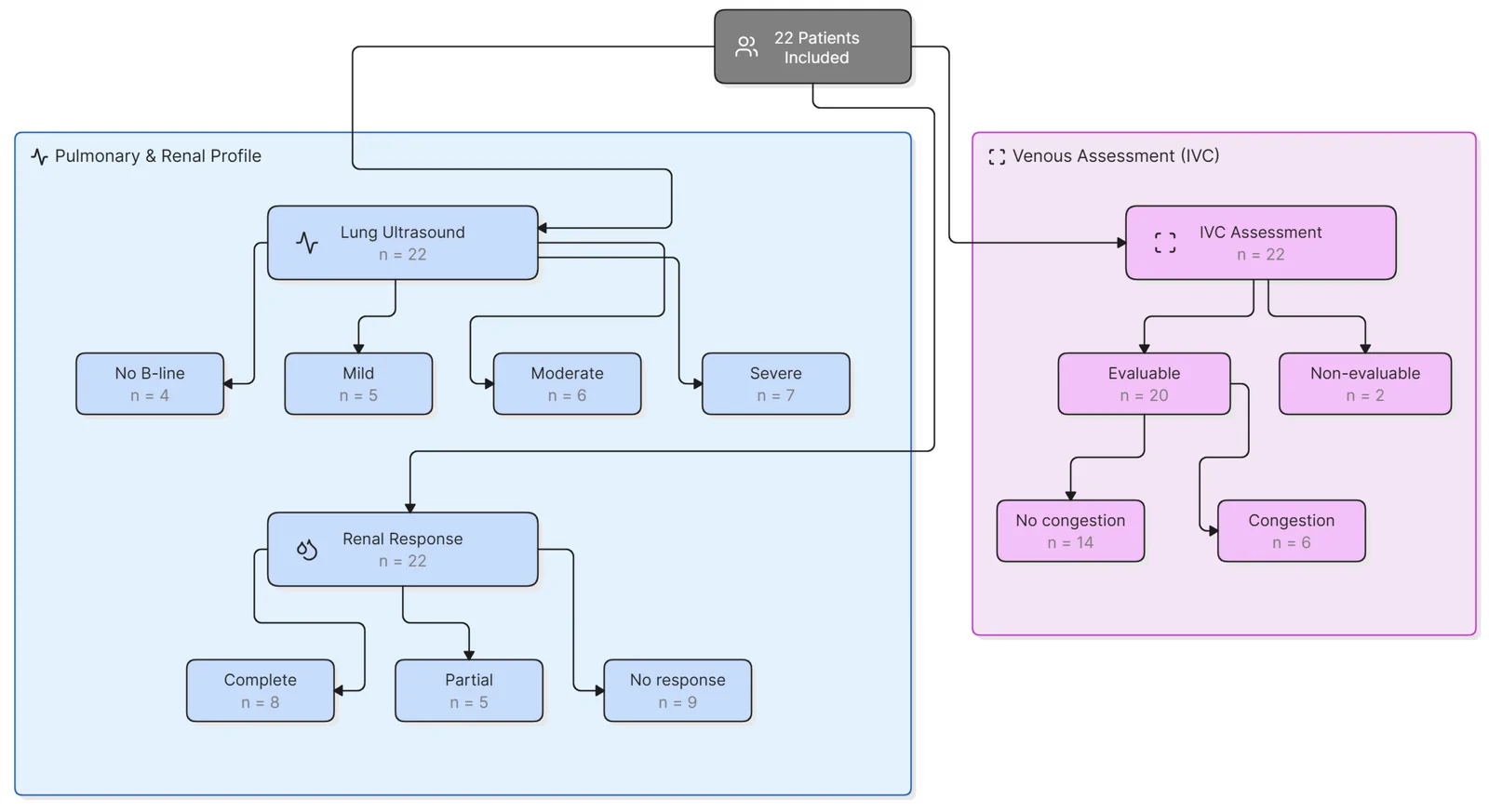
Participant flow and evaluable POCUS domains. The figure was created using the web application eraser.io.

A total of 22 patients with liver cirrhosis and AKI who were eligible for intravenous albumin administration met all inclusion criteria and no exclusion criteria. All 22 patients were included in the pulmonary congestion analysis and renal follow-up analysis. No participants were lost during follow-up, and no deaths occurred during the study period. Inferior vena cava assessment was technically feasible in 20 patients. In 2 patients, venous congestion could not be evaluated because the inferior vena cava could not be reliably visualized due to anatomical limitations or inadequate acoustic window (Figure 1). The mean age of the cohort was 51.09 ± 13.216 years, and most patients were male (*n* = 18, 82%). The most frequent etiology of liver cirrhosis was alcohol-related liver disease (*n* = 8, 36%). The most common admission diagnosis was AKI with ascites (*n* = 10, 46%), followed by acute exacerbation of chronic liver failure (*n* = 6, 27%). The baseline sociodemographic characteristics, clinical history, etiology of cirrhosis, and admission diagnoses are summarized in Table 1.

Based on POCUS evaluation, pulmonary congestion was identified in 18 of 22 patients (81.8%). Among patients with pulmonary congestion, severity was classified as mild in 27.8%, moderate in 33.3%, and severe in 38.9%. Table 2 presents serum creatinine values before and 48 h after albumin administration according to the presence or absence of pulmonary congestion.

Among patients without B-line-defined pulmonary congestion, mean serum creatinine was 2.27 ± 0.65 mg/dL before albumin administration and 2.48 ± 1.04 mg/dL after 48 h. The Hodges–Lehmann estimate of the median paired difference was +0.03 mg/dL (95% CI, −0.52 to 1.31; exact *p* = 0.875). Among patients with B-line-defined pulmonary congestion, mean serum creatinine decreased from 2.26 ± 0.89 mg/dL to 1.80 ± 0.99 mg/dL. The estimated median paired difference was −0.33 mg/dL (95% CI, −0.67 to −0.20; exact *p* = 0.008). Renal response occurred in 1 of 4 patients without pulmonary congestion (25.0%) and in 12 of 18 patients with pulmonary congestion (66.7%); the difference in responder proportions was not statistically significant (two-sided Fisher exact *p* = 0.264). These analyses are exploratory and do not demonstrate a differential effect of albumin according to baseline pulmonary congestion status.

Serum creatinine values were summarized descriptively according to pulmonary congestion severity. Mean serum creatinine changed from 2.27 ± 0.65 mg/dL to 2.48 ± 1.04 mg/dL in patients without pulmonary congestion, from 2.51 ± 1.19 mg/dL to 1.74 ± 0.67 mg/dL in those with mild congestion, from 2.05 ± 0.79 mg/dL to 1.27 ± 0.42 mg/dL in those with moderate congestion, and from 2.28 ± 0.84 mg/dL to 2.30 ± 1.34 mg/dL in those with severe congestion. Because subgroup sizes ranged from four to seven patients, no formal statistical comparisons were performed, and the observed patterns should be interpreted as exploratory and hypothesis-generating (Table 3).

Renal response occurred in 1 of 4 patients without pulmonary congestion (25.0%), 3 of 5 with mild congestion (60.0%), 5 of 6 with moderate congestion (83.3%), and 4 of 7 with severe congestion (57.1%). These subgroup proportions are presented descriptively without formal hypothesis testing.

Inferior vena cava assessment was technically feasible in 20 of 22 patients. IVC-defined venous congestion was identified in 6 of the 20 evaluable patients (30.0%), whereas 14 (70.0%) did not meet the prespecified IVC criteria. In patients without IVC-defined venous congestion, mean serum creatinine was 2.38 ± 0.92 mg/dL before albumin and 1.97 ± 0.82 mg/dL after 48 h; the Hodges–Lehmann estimate was −0.23 mg/dL (95% CI, −0.90 to 0.35; exact *p* = 0.123). In patients with IVC-defined venous congestion, mean serum creatinine was 2.22 ± 0.74 mg/dL before albumin and 2.15 ± 1.44 mg/dL after 48 h; the estimate was −0.33 mg/dL (95% CI, −0.60 to 1.60; exact *p* = 0.438). Renal response occurred in 8 of 14 patients without IVC-defined venous congestion (57.1%) and in 3 of 6 patients with IVC-defined venous congestion (50.0%; two-sided Fisher exact *p* = 1.000). The two patients with non-evaluable IVC examinations remained included in pulmonary and overall renal-response analyses (Table 4).

No clear differences in renal response proportions were observed according to pulmonary or venous congestion status. However, POCUS identified a high frequency of pulmonary congestion and a lower but clinically relevant frequency of venous congestion in this cohort of patients with cirrhosis and AKI receiving albumin therapy.

## 4. Discussion

In this exploratory prospective study, baseline POCUS frequently identified B-line-defined pulmonary congestion and, less frequently, IVC-defined venous congestion in patients with cirrhosis and AKI receiving albumin therapy. However, baseline congestion status did not clearly distinguish patients according to short-term renal response. These findings support POCUS as a bedside phenotyping tool but do not establish its ability to predict renal recovery, guide albumin administration, or determine clinical outcomes.

The high B-line burden observed in this cohort is consistent with previous reports in patients with AKI, in whom lung ultrasound may identify sonographic abnormalities not apparent on conventional clinical examination [19,20]. B-lines may accompany increased extravascular lung water but are not specific for cardiogenic pulmonary edema, elevated cardiac filling pressures, or generalized volume overload. In patients with cirrhosis and AKI, they may reflect cardiogenic mechanisms, non-cardiogenic lung water related to inflammation or endothelial dysfunction, or mixed mechanisms [21,22,23,24]. Therefore, the baseline B-line burden observed in this study should not be interpreted in isolation as clinically confirmed pulmonary edema, as a contraindication to albumin therapy, or as evidence of albumin-related harm.

The descriptive analysis by pulmonary congestion severity showed different creatinine trajectories across the small subgroups. Mean creatinine changed minimally in the severe category, whereas lower values at 48 h were observed in the mild and moderate categories. This pattern may reflect heterogeneity in baseline liver disease severity, renal injury phenotype, cardiac function, or systemic inflammation; it may also represent random variability arising from subgroup sizes of only four to seven patients. Because cardiac hemodynamics, liver severity scores, and post-albumin POCUS were unavailable, the study cannot determine whether a cardiogenic component or a more advanced hemodynamic phenotype explains this observation. No inferential conclusion should therefore be drawn from the severity categories.

Albumin has hemodynamic and non-hemodynamic effects that may be relevant in cirrhosis-associated AKI, including plasma expansion, circulatory support, endothelial stabilization, binding of inflammatory mediators, and modulation of systemic inflammation [25,26]. The lack of clear separation in renal response according to baseline B-line or IVC status is compatible with the multifactorial nature of AKI in cirrhosis and the influence of unmeasured clinical factors. Renal response is unlikely to depend solely on one baseline ultrasound parameter.

IVC-defined venous congestion was present in a smaller proportion of technically evaluable patients. Prior studies have shown that elevated venous pressures or cardiac dysfunction may be underrecognized in patients initially classified as having hepatorenal physiology [13,27,28]. Nevertheless, IVC diameter and collapsibility may be influenced by the hyperdynamic circulation of cirrhosis, ascites, intra-abdominal pressure, respiratory mechanics, and technical acoustic limitations. The present study did not include a multiorgan venous congestion protocol or invasive hemodynamic confirmation; therefore, IVC findings should be interpreted as an operational ultrasound classification rather than a comprehensive diagnosis of systemic venous congestion.

The principal clinical contribution of this study is the demonstration that baseline pulmonary and IVC ultrasound assessment was feasible in a complex hospitalized population. POCUS may complement, but not replace, clinical judgment. Future studies should incorporate serial POCUS before and after albumin administration, standardized treatment documentation, formal cardiac assessment, liver disease severity scores, detailed AKI phenotyping, and clinically meaningful renal, respiratory, and survival outcomes.

Several limitations should be acknowledged. First, the sample was small, and the comparison groups were markedly unbalanced, limiting statistical precision, power, and generalizability. Although exact methods and confidence intervals were used, all comparative analyses remain exploratory and hypothesis-generating. Second, important variables were unavailable or incompletely recorded, including Child–Pugh class, MELD score, ACLF grade, detailed infection and spontaneous bacterial peritonitis status, mean arterial pressure, serum sodium, bilirubin, INR, serum albumin, urine output, vasoconstrictor use, fluid balance, beta-blocker withdrawal, diuretic withdrawal, and the exact administered albumin dose and infusion volume. The absence of Child–Pugh and MELD data is particularly important because baseline liver disease severity may influence both the congestion phenotype and the likelihood of renal recovery; consequently, the observed creatinine changes cannot be causally attributed to baseline POCUS findings or albumin administration. Third, 64% of participants had received loop diuretics, but the timing of withdrawal relative to POCUS was not consistently documented. Treatment-related effects may therefore have influenced B-lines and IVC measurements, and the observed findings cannot be assumed to represent an untreated volume state. Fourth, POCUS was performed only before albumin administration; the study cannot determine whether albumin caused, worsened, or improved pulmonary or venous congestion. Fifth, IVC assessment was not technically feasible in two patients. Sixth, examinations were performed by one operator, which improved procedural consistency but precluded assessment of interobserver variability. Finally, the study lacked long-term follow-up and was not designed or powered to evaluate the effectiveness or safety of albumin therapy.

Despite these limitations, the study provides preliminary information on baseline POCUS phenotypes in cirrhosis-associated AKI. The results should not be interpreted as evidence for or against albumin effectiveness in patients with ultrasound-defined congestion. Rather, they support further prospective evaluation of POCUS-informed clinical characterization using serial measurements and adequately powered outcomes.

## 5. Conclusions

In this exploratory prospective study conducted at a tertiary referral center in western Mexico, baseline POCUS frequently identified B-line-defined pulmonary congestion and identified IVC-defined venous congestion in a smaller proportion of technically evaluable patients with cirrhosis and AKI receiving albumin therapy. Baseline congestion status did not clearly distinguish patients according to short-term renal response. Because POCUS was performed only before albumin administration, these findings cannot determine whether albumin caused, worsened, or improved congestion, whether congestion modified the renal response to albumin, or whether POCUS-guided management improves clinical outcomes. The results support the feasibility of bedside POCUS phenotyping and should be confirmed in larger prospective studies incorporating standardized treatment documentation, serial POCUS assessments, liver disease severity scores, and clinically meaningful renal, respiratory, and survival outcomes.

## Figures and Tables

**Table 1:** Sociodemographic characteristics and clinical history of the study population.

Patient Characteristics	Total Number of Patients = 22
Age, years, mean ± SD	51.09 ± 13.216
Sex, *n* (%)	
Male	18 (82)
Clinical history, *n* (%)	
Cardiac disease	0 (0)
Loop diuretic administration	14 (64)
Crystalloid administration	4 (18)
Etiology of liver cirrhosis, *n* (%)	
Autoimmune hepatitis	4 (18)
Non-alcoholic fatty liver disease	4 (18)
Alcohol-related liver disease	8 (36)
Indeterminate	3 (14)
Hepatitis C virus	2 (9)
Hepatitis B virus	1 (5)
Admission diagnosis, *n* (%)	
Hepatic encephalopathy	3 (14)
Acute kidney injury and ascites	10 (46)
Non-active gastrointestinal bleeding	1 (5)
Acute exacerbation of chronic liver failure	6 (27)
Spontaneous bacterial peritonitis	2 (9)

SD = standard deviation.

**Table 2:** Serum creatinine and renal response according to baseline B-line-defined pulmonary congestion status.

Baseline Pulmonary Status	Pre-Albumin Creatinine, Mean ± SD (mg/dL)	48-h Creatinine, Mean ± SD (mg/dL)	Hodges–Lehmann Paired Difference (95% CI), mg/dL	Exact Wilcoxon *p*	Renal Responders, *n* (%)
No B-line-defined congestion (*n* = 4)	2.27 ± 0.65	2.48 ± 1.04	+0.03 (−0.52 to 1.31)	0.875	1 (25.0)
B-line-defined congestion (*n* = 18)	2.26 ± 0.89	1.80 ± 0.99	−0.33 (−0.67 to −0.20)	0.008	12 (66.7)

SD = standard deviation; CI = confidence interval. The difference in renal responder proportions was evaluated using the two-sided Fisher exact test (*p* = 0.264).

**Table 3:** Descriptive summary of serum creatinine and renal response according to pulmonary congestion status and severity.

Pulmonary Congestion Category	Pre-Albumin Creatinine, Mean ± SD (mg/dL)	48-h Creatinine, Mean ± SD (mg/dL)	Renal Responders, *n* (%)
No congestion (*n* = 4)	2.27 ± 0.65	2.48 ± 1.04	1 (25.0)
Mild congestion (*n* = 5)	2.51 ± 1.19	1.74 ± 0.67	3 (60.0)
Moderate congestion (*n* = 6)	2.05 ± 0.79	1.27 ± 0.42	5 (83.3)
Severe congestion (*n* = 7)	2.28 ± 0.84	2.30 ± 1.34	4 (57.1)

SD = standard deviation. Owing to the small subgroup sizes (*n* = 4–7), the data are presented descriptively without formal hypothesis testing.

**Table 4:** Serum creatinine and renal response according to baseline IVC-defined venous congestion status among patients with technically evaluable IVC examinations.

Baseline IVC Status	Pre-Albumin Creatinine, Mean ± SD (mg/dL)	48-h Creatinine, Mean ± SD (mg/dL)	Hodges–Lehmann Paired Difference (95% CI), mg/dL	Exact Wilcoxon *p*	Renal Responders, *n* (%)
No IVC-defined congestion (*n* = 14)	2.38 ± 0.92	1.97 ± 0.82	−0.23 (−0.90 to 0.35)	0.123	8 (57.1)
IVC-defined congestion (*n* = 6)	2.22 ± 0.74	2.15 ± 1.44	−0.33 (−0.60 to 1.60)	0.438	3 (50.0)

SD = standard deviation; CI = confidence interval. Venous analyses included 20 patients. The difference in renal responder proportions was evaluated using the two-sided Fisher exact test (*p* = 1.000). Two patients with non-evaluable IVC examinations remained included in pulmonary and overall renal-response analyses.

## Data Availability

The data presented in this study are available on request from the corresponding author due to privacy reasons.
